# Cancer of the Left Mammary Gland, Followed by Tetanus

**Published:** 1882-06

**Authors:** Charles B. Ziegler

**Affiliations:** Baltimore


### CLINICAL REPORTS.

### I. CANCER OF THE LEFT MAMMARY GLAND FOLLOWED BY TETANUS.—II. CANCER OF THE ANTRUM.

BY CHARLES B. ZIEGLER, M.D., BALTIMORE,
[Read before the Baltimore Medical and Surgical Society.]

Case I. — On the 20th day of August, 1878, a mulatto woman, aged 40, called to see me with reference to what she called a sore on her left breast. Upon questioning her, she stated that about six or seven months before she had had severe shooting pains, as if a knife had been darted through her side. These pains centred in the left breast. Later she discovered a small hard "lump'' in the breast, a little to the left of the nipple. This became larger, and the nipple, she said, seemed to "fall in.''

About six weeks before she came to see me, the skin became inflamed and ulcerated, discharging a thin and offensive matter.

Upon examination of the breast, I found an ulcer measuring about an inch and a half in diameter, situated to the left of the nipple. This ulcer had hard ragged edges, elevated about one third of an inch above the surrounding skin. These edges presented a knobby and fissured aspect, the fissures becoming broader and deeper until they were blended into the general excavation of the ulcer. To the touch the base was hard and nodulated. The surface of the ulcer was covered by an ash-colored exudation.

The pain of which she complained at this time was of a burning and gnawing character, with occasional shooting pains. She was anemic, and was very much distressed about her condition, fearing the worst. She could not remember having received any injury to the breast, but had had mastitis. She had borne but one child, who was about eight

---

years of age at the time the case came under observation. There was, as far as she knew, no one of her family who had suffered from cancerous growths of any kind.

As the symptoms, both objective and subjective, pointed to malignant growth, and as they were for the most part parallel to those of scirrhus of the breast, I pronounced it such ; and as the glands in the axilla were not affected, I advised an early operation. Upon the mention of an operation she became very nervous, so nervous indeed that she was taken with perceptible trembling and for a few minutes could scarcely speak. I therefore did not press the subject, but prescribed an anodyne to relieve the pain that she had complained of, and told her to decide, and let me know of her decision later, advising her, however, not to put it off too long.

On the 23d of August, three days after the above consultation, her husband called upon me, requesting me to see his wife, stating that she was suffering a great deal of pain, and that she could not open her mouth. I went immediately, and found, as her husband had told me, that her jaws were firmly clinched, and that she suffered great pain from spasm of the muscles of mastication. Later in the day, spasm of the muscles of the upper and lower extremities came on. Slight disturbances seemed to cause her great pain, and the contractions increased so that her limbs became hard and rigid. The muscles of the trunk were but little affected, and consequently there was no apparent opisthotonus. The muscles of deglutition were early affected, so that it was almost impossible to give anything by the mouth. Her condition became progressively worse, and she died on the 25th, two days later.

Case II.—Wm. R. called upon me during the month of October, 1879, and gave the following history—viz.: Was nearly forty-eight years of age, by occupation a farm-hand, but had also been a sailor. He was intemperate in his habits, but had never contracted syphilis. During July and August he had suffered from occasional pains in the left jaw, which he then attributed to neuralgia. These pains became of more frequent occurrence, and much more severe during September, but he bore with them, without seeking advice, until the first week in October, when, thinking that a decayed first molar tooth was the cause of the trouble, he had it drawn by the village dentist. The pain, however, instead of being relieved, grew worse, and he was forced to seek surgical advice. It was at this time that he came under my observation.

Upon examination I found that a thick, putty-like matter filled the socket of the extracted tooth. Making use of a probe, I found that this cavity communicated with that of the antrum. The hard palate was somewhat depressed on the affected side. The cheek was swollen, but not tender. The teeth on that side, he complained, felt longer than those of the opposite side, and there was a discharge of pus from the nostril.

I diagnosticaed abscess of the antrum, and ordered injections of warm water, carbolized, to be used two or three times a day. The nozzle of the syringe was introduced at the molar opening, which had been dilated, and the water was forced into the antrum, from which it flowed into and from the nostrils. I also prescribed tonics, and advised a good nutritious diet. Under this treatment he improved ; but this apparent improvement was only temporary.

About the last of November a small body, apparently composed of granulations, projected from the socket of the extracted tooth, and shortly afterward the remaining teeth on that side became loose and literally dropped out, and the sockets were found to be occupied by a continuation of this body. It grew rapidly, and soon filled his mouth to such an extent that it was only with difficulty that he was able to bring his lips together, they remaining when at rest about half an inch apart. The body was soft, feeling much like brain substance when taken between the fingers, and bled from very slight causes—in fact, he had two severe hemorrhages from it during the course of the disease. The discharge was considerable, and very offensive.

The pain that he now complained of was of a grinding character. He could not sleep on account of it, and anodynes in large doses gave but little relief. This pain, however, gradually became less severe, and finally gave him but little uneasiness. But he still complained of constant aching of the jaw ; not violent, but sufficient to annoy him.

His condition in January, 1880, showed local symptoms worse. His general condition was also bad. The palate was much more depressed, the cheek swollen very much, and tender, and the eye protruded slightly.

In February an opening occurred through the cheek, which was almost from the first surrounded by large fungous granulations, that soon covered the cheek to the extent of the surface of a silver half-dollar.

At this time the patient presented a most unsightly appearance. The eye protruded forward ; the cheek was greatly swollen, with the fungous granulations for a cap; the lips widely apart, and the saliva mixed with blood and pus running from his mouth, constituted a sad picture of suffering. His suffering was not to last much longer, for about the last of February he became dull and hard of hearing; he slept much, became semi-comatose, and after a few days fell into a completely comatose condition, and died during the first week in March, 1880.

I will here state that my first diagnosis of abscess was changed to that of cancer upon the development of symptoms showing the malignancy of the disease.

